# CCL5 Induces a Sarcopenic-like Phenotype via the CCR5 Receptor

**DOI:** 10.3390/antiox14010084

**Published:** 2025-01-13

**Authors:** Francisco Aguirre, Franco Tacchi, Mayalen Valero-Breton, Josué Orozco-Aguilar, Sabrina Conejeros-Lillo, Josefa Bonicioli, Renata Iturriaga-Jofré, Daniel Cabrera, Jorge A. Soto, Mauricio Castro-Sepúlveda, Marianny Portal-Rodríguez, Álvaro A. Elorza, Andrea Matamoros, Felipe Simon, Claudio Cabello-Verrugio

**Affiliations:** 1 Laboratory of Muscle Pathology, Fragility and Aging, Department of Biological Sciences, Faculty of Life Sciences, Universidad Andres Bello, Santiago 8370146, Chile; f.aguirregalaz@uandresbello.edu (F.A.); f.tacchifernandez@uandresbello.edu (F.T.); m.valerobreton@uandresbello.edu (M.V.-B.); josue.orozco@ucr.ac.cr (J.O.-A.); sabrina.conejeros@gmail.com (S.C.-L.); j.bonicioliprez@uandresbello.edu (J.B.); r.iturriagajofr@uandresbello.edu (R.I.-J.); m.portalrodriguez@uandresbello.edu (M.P.-R.); 2 Millennium Institute on Immunology and Immunotherapy, Faculty of Life Sciences, Universidad Andres Bello, Santiago 8370146, Chile; jorge.soto.r@unab.cl; 3 Centro de Investigación e Innovación Biomédica (CiiB), Universidad de los Andes, Santiago 7620001, Chile; dacabrera@uandes.cl; 4 Facultad de Ciencias de la Salud, Escuela de Kinesiología, Universidad Bernardo O Higgins, Santiago 8370993, Chile; 5 Translational Immunology Laboratory, Department of Biological Sciences, Faculty of Life Sciences, Universidad Andres Bello, Santiago 8370146, Chile; 6 Exercise Physiology and Metabolism Laboratory, School of Kinesiology, Faculty of Medicine, Finis Terrae University, Santiago 7501014, Chile; mcastro@uft.cl; 7 Institute of Biomedical Sciences, Faculty of Medicine and Faculty of Life Sciences, Universidad Andres Bello, Santiago 8370071, Chile; alvaro.elorza@unab.cl (Á.A.E.); a.matamorospalacios@uandresbello.edu (A.M.); 8 Laboratory of Integrative Physiopathology, Department of Biological Sciences, Faculty of Life Sciences, Universidad Andres Bello, Santiago 8370146, Chile

**Keywords:** sarcopenia, CCL5, CCR5, oxidative stress, ubiquitin–proteasome system, protein synthesis, gene electroporation, reactive oxygen species

## Abstract

Sarcopenia corresponds to a decrease in muscle mass and strength. CCL5 is a new myokine whose expression, along with the CCR5 receptor, is increased in sarcopenic muscle. Therefore, we evaluated whether CCL5 and CCR5 induce a sarcopenic-like effect on skeletal muscle tissue and cultured muscle cells. Electroporation in the tibialis anterior (TA) muscle of mice was used to overexpress CCL5. The TA muscles were analyzed by measuring the fiber diameter, the content of sarcomeric proteins, and the gene expression of E3-ligases. C_2_C_12_ myotubes and single-isolated flexor digitorum brevis (FDB) fibers were also treated with recombinant CCL5 (rCCL5). The participation of CCR5 was evaluated using the antagonist maraviroc (MVC). Protein and structural analyses were performed. The results showed that TA overexpression of CCL5 led to sarcopenia by reducing muscle strength and mass, muscle-fiber diameter, and sarcomeric protein content, and by upregulating E3-ligases. The same sarcopenic phenotype was observed in myotubes and FDB fibers. We showed increased reactive oxygen species (ROS) production and carbonylated proteins, denoting oxidative stress induced by CCL5. When the CCR5 was antagonized, the effects produced by rCCL5 were prevented. In conclusion, we report for the first time that CCL5 is a novel myokine that exerts a sarcopenic-like effect through the CCR5 receptor.

## 1. Introduction

Skeletal muscle, the most abundant tissue in the body, performs various functions through muscle contraction [1]. The sarcomere is the functional unit of skeletal muscle, which contains proteins that participate in the contraction process, such as the α-actin, myosin heavy chain (MHC), and troponin [2].

Among numerous disorders that can change the physiology of the muscle is sarcopenia, which corresponds to a decline in muscle strength and mass, leading to impaired physical function [3]. Sarcopenia is associated with reduced muscle-fiber diameter and content of sarcomeric proteins concomitant with an imbalance in the protein synthesis and degradation mechanisms [4]. Specifically, protein degradation is related to the ubiquitin–proteasome system (UPS) by activating E3 ligases MuRF-1 and atrogin-1. Oxidative stress has also been reported as an underlying mechanism of sarcopenia [5,6], with increased reactive oxygen species (ROS) as a feature of elevated oxidant status [7,8].

Furthermore, soluble factors, some of which can induce sarcopenia, influence muscle physiology in a paracrine and autocrine fashion [9]. Chemokines and myokines, often associated with inflammatory processes, are associated with sarcopenia and other biological processes, including angiogenesis, protease secretion, and differentiation [10]. CCL5 is a chemokine expressed by various cell types, including skeletal muscle [11,12,13]. Elevated levels of CCL5 have been observed in muscular inflammatory processes [14]. In contrast, CCL5 decreases its expression in skeletal muscle in response to contractile activity [15]. Skeletal muscle expresses two CCL5 receptors, CCR1 and CCR5 [16]. Furthermore, we show that ccl5 mRNA levels increase in skeletal muscle in a model of sarcopenia due to cholestatic liver disease and induce some alterations in sarcomeric proteins related to UPS [12,17]. Despite these previous findings, the direct sarcopenic effect of CCL5 on skeletal muscle and the receptor involved remains unclear.

Therefore, in this research, we sought to evaluate the sarcopenic role of CCL5 overexpression in skeletal muscle. We also determined the ability of CCL5 to produce a sarcopenic-like effect in C_2_C_12_ myotubes and single-isolated muscle fibers through the CCR5 receptor.

Our research demonstrates that the overexpression of CCL5 in the tibialis anterior (TA) muscle induces sarcopenia, reducing muscle mass and strength. This overexpression also results in a consistent decrease in fiber diameter and sarcomeric protein content. Moreover, CCL5 appears to upregulate components of the UPS and, through the CCR5 receptor, induces a sarcopenic-like phenotype in C_2_C_12_ myotubes and isolated FDB muscle fibers. This pathway disrupts the balance between protein synthesis and degradation by increasing the expression of E3 ligases and decreasing protein synthesis rates. Additionally, CCL5, via CCR5, elevates oxidative stress via increased ROS production and carbonylated protein in skeletal muscle cells.

## 2. Materials and Methods

### 2.1. In Vivo Gene Transfer and Electroporation

C57BL6 male mice (3–4 months old) were injected intramuscularly with hyaluronidase into the tibialis anterior (TA) muscle for 1 h. To overexpress CCL5, an injection of 30 µg of a plasmid carrying mouse cDNA for CCL5 and green fluorescent protein (GFP) (VectorBuilder Inc. VB211006-1283atd, Chicago, IL, USA) (oeCCL5) under control of independent promoters, or the plasmid only with the cDNA sequence for GFP used as a control (Mock) (VectorBuilder Inc. VB010000-9288rhy, Chicago, IL, USA), was administered. A current was applied to the TA muscle by eight pulses of 20 ms duration every 230 ms at 200 V/cm using ECM830 square pulse electroporation equipment (BTX Harvard Apparatus, Holliston, MA, USA). Mice were sacrificed seven and twenty-one days after electroporation, and the TA muscles were removed to extract proteins and RNA, obtain histological sections, and determine isometric strength.

### 2.2. Determination of Isometric Strength of the Isolated Tibialis Anterior (TA) Muscle

The contractile properties were measured in isolated TA muscles subjected to electrostimulation. Briefly, TA muscles were removed from mice by cutting off the tendon from an extreme and leaving the joint to the patella from another extreme. Further, TA was attached to a force transducer (Power Lab 4/35, AD Instruments, Colorado Springs, CO, USA). Thus, the TA muscle was fixed from no-muscular tissue and immersed in an oxygenated Krebs–Ringer solution. Micromanipulation of the muscle length was used to determine the maximum length (Lo) to produce the maximum isometric force. The muscle was subjected to stimuli of different frequencies between 1 and 150 Hz, 450 ms duration, with 2 min of rest between each stimulus using a stimulator (Grass S48 Stimulator, Grass Instruments, Astro-Med, Inc. West Warwick, RI, USA), and it showed a curve frequency vs. force. The specific net force was calculated and normalized by the tibia length and expressed as mN/cm. as described above [18,19,20,21,22].

### 2.3. Muscle-Fiber Diameter Determination

Cryosections (10 µm) of TA were incubated with an anti-laminin antibody (1:100 Santa Cruz Biotechnology catalog #sc-59854, Dallas, TX, USA), and immunofluorescent staining was performed to visualize the fiber diameter. The Motic BA310 epifluorescence microscope (Motic, Hong Kong) was employed for visualization, and the minimal Feret diameters were quantified using ImageJ software (NIH, Bethesda, MD, USA).

### 2.4. C_2_C_12_ Cell Culture

The C_2_C_12_ skeletal muscle cell line (ATCC, Manassas, VA, USA) was cultured in a growth medium (DMEM supplemented with 10% fetal bovine serum) at 37 °C and 5% CO_2_ until reaching 90% confluence. DMEM supplemented with 4% horse serum was used to induce differentiation into myotubes. On the fifth day of differentiation, myotubes were incubated with 50, 100, or 200 ng/mL of human recombinant CCL5 (rCCL5) (ProSpec catalog #chm-328-b, East Brunswick, NJ, USA) for the specified time indicated in each experiment. To assess the role of the CCR5 receptor, myotubes were pre-incubated with 10 µM of maraviroc (MVC) (Tocris catalog #3756, Minneapolis, MN, USA) and 10 μM of J113863 (Tocris catalog #2595, Minneapolis, MN, USA), which are CCR5 and CCR1 pharmacological antagonist, respectively, for 1 h before exposure to rCCL5, whose concentration has been previously reported [23]. 

### 2.5. Cell Viability MTT

The myotubes of C_2_C_12_ were incubated with several concentrations (0, 10, 50, 100, 200, and 400 ng/mL) of rCCL5 for 72 h. At the end of the experiment, 10 µL of MTT solution (5 mg/mL, pH 7.5) was added. After 1 h, blue formazan crystals were diluted with 100 µL DMSO. Finally, we measured the absorbance at 595 nm. 

### 2.6. Single-Isolated Muscle-Fiber Culture

Single-isolated fibers were obtained from the flexor digitorum brevis (FDB) muscle of C57BL/6 male mice (3–4 months old) through enzymatic digestion of the whole muscle using 375 U/mL of collagenase-type I (Worthington catalog #LS004197, Lakewood, NJ, USA) in DMEM for 90 min at 37 °C. Following muscle digestion, the medium with collagenase was removed, and DMEM supplemented with 10% horse serum was added and mechanically dissociated by passage through fire-polished Pasteur pipettes. Approximately 50 single-isolated fibers were transferred into Matrigel-coated 24-well plates. We let it adhere for 24 h and incubated with rCCL5 at 200 ng/mL. Pre-incubation with 10 µM of MVC and J113863, respectively, was performed 1 h before exposure to rCCL5.

### 2.7. Western Blot Analysis

TA muscles and myotubes were homogenized using a radioimmunoprecipitation assay buffer containing phosphatase inhibitors (1 mM sodium orthovanadate and 1 mM sodium pyrophosphate), as well as a cocktail of protease inhibitors (1 mM) and phenylmethylsulfonyl fluoride (1 mM). The protein extracts were separated by SDS-PAGE and were transferred onto a polyvinylidene difluoride membrane. Immunoblotting was carried out using the following primary antibodies: mouse anti-MHC (1:1000 MF-20; Developmental Studies Hybridoma Bank, University of Iowa, IA, USA), mouse anti-troponin I (1:1000; Cell Signaling, Danvers, MA, USA), mouse anti-αTubulin (1:1000; Santa Cruz Biotechnology catalog #3873S, Dallas, TX, USA), mouse anti-β-actin (1:2000, Abcam catalog #3700T, Cambridge, MA, USA), mouse anti-puromycin (EMD Millipore catalog #MABE343, Burlington, MA, USA), rabbit anti-tropomyosin-1 (1:1000; Cell Signaling catalog #3910S, Danvers, MA, USA), rabbit anti-CCR1 (1:1000; NOVUS Biologicals, cat#NB100-56334, Easter Ave Centennial, CO, USA), mouse anti-CCR5 (1:1000; Invitrogen, cat#PA5-114965 Waltham, MA, USA), and rabbit anti-CCL5 (1:300, Cell Signaling catalog #36467S). Subsequently, the following appropriate secondary antibodies were used: anti-mouse IgG-HRP (1:10,000; Santa Cruz Biotechnology, Dallas, TX, USA) and anti-rabbit IgG-HRP (1:10,000; Santa Cruz Biotechnology, Dallas, TX, USA). The immunoreaction was visualized through enhanced chemiluminescence (Thermo Scientific, Waltham, MA, USA). Images were captured using the Fotodyne FOTO/Analyst Luminary Workstation Systems (Fisher Scientific, Waltham, MA, USA), and band quantification was performed using densitometric analysis with ImageJ software (NIH, Bethesda, MD, USA).

### 2.8. RNA Extraction and RT-qPCR

RNA extraction from the TA muscles and myotubes was conducted using Chomczynski’s solution (a phenol–chloroform-based solution). Two μg of RNA was used for reverse transcription, and cDNA was synthesized using the M-MLV enzyme. Subsequent qPCR was performed in the Eco Real-Time PCR System (Illumina catalog #1010180, San Diego, CA, USA). The genes *murf-1* (*F: GCTGGTGGAAAACATCATTGACAT*, *R: CATCGGGTGGCTGCCTTT*), *atrogin-1* (*F: CACATTCTCTCCTGGAAGGGC*, *R: TTGATAAAGTCTTGAGGGGAAAGTG*), *ccl5* (*F: ATCTCTGCAGCTGCCCTCAC*, *R: CTTGGCGGTTCCTTCGAGTG*), *ccr5* (*F: TTCTTCTGTGGATCGGGTATAGA*, *R: TTATCTCTCAGTGTTCTTCCGAAAAC*), *ccr1* (*F: AGGCCCAGTGGGAGTTCA*, *R: TCTTCCACTGCTTCAGGCTCTT*), *gfp* (*F: CTGACCCTGAAGTTCATCTG*, *R: GAAGTCGTGCTGCTTCAT*), and *β-actin* (*F: GTGACGTTGACATCCGTAAAGA*, *R: GCCGGACTCATCGTACTCC*) were assayed. The mRNA expression of target genes was calculated using the ∆∆Ct method, normalized relative to *β-actin* gene expression.

### 2.9. Immunofluorescence Microscopy

Myotubes and single-isolated FDB fibers were incubated with 200 ng/mL of rCCL5 for the specified time indicated in each experiment. Myotubes and single-isolated fibers were fixed with 4% paraformaldehyde (PFA) for 5 min. After fixation, the sample was washed twice with PBS 1X and permeabilized with 0.05% Triton X-100 for 10 min. Twice, the wash was repeated and blocked with 1% BSA for 1 h. The samples were incubated with the following primary antibodies: mouse anti-Caveolin-3 (1:500, Santa Cruz catalog #SC-5310 Biotechnology, Dallas, TX, USA), rabbit anti-GFP (1:100, Invitrogen catalog #A11120, Waltham, MA, USA), and mouse anti-puromycin (1:1000, EMD Millipore catalog #MABE343, Burlington, MA, USA) in PBS-1% BSA. After the incubation, an affinity-purified Alexa Fluor dye-conjugated goat anti-mouse antibody was applied (1:200, Life Technologies catalog #A11001(green) and A11004 (red), Waltham, MA, USA), and an affinity-purified Alexa Fluor dye-conjugated goat anti-rat antibody was also applied (1:200, Life Technologies catalog#A11007, Waltham, MA, USA). Samples were observed using a Motic BA310 epifluorescence microscope (Motic, Hong Kong).

### 2.10. C_2_C_12_ Myotubes and Single-Isolated FDB Fiber Diameter Determination

Myotubes and single-isolated FDB fibers were incubated with rCCL5 at 200 ng/mL for 72 h. Indirect immunofluorescence for caveolin-3 was conducted, and the analysis was carried out using ImageJ software through the measurement of Feret minimum (NIH, Bethesda, MD, USA). The diameter of C_2_C_12_ myotubes and isolated muscle fibers from the FDB was assessed across three independent biological replicates (*n* = 3). Each “*n*” corresponds to a cell culture from different passages of C_2_C_12_ myotubes. A total of 80 myotubes were evaluated per condition for each experimental “*n*”, resulting in approximately 240 myotubes analyzed per condition across all three replicates. Regarding isolated fibers, the FDB muscles were extracted from the hindlimb of three mice. Each pair of FDB muscles from each mouse was considered a biological replicate, and 50 isolated fibers were measured per condition for each experimental “*n*”, culminating in the analysis of 150 isolated FDB fibers per condition across three replicates.

### 2.11. SUrface SEnsing of Translation (SUnSET) Assay

Myotubes and single-isolated FDB fibers were pre-incubated for 24 h with 1 mM puromycin (Sigma catalog #P8833) and then incubated with rCCL5 at 200 ng/mL for 24 h. Western blot and indirect immunofluorescence were performed employing an antibody to detect puromycin incorporation and were analyzed using ImageJ (NIH, Bethesda, MD, USA). Puromycin intensities were determined as the fluorescence intensity for each fiber. The incorporation of puromycin in isolated muscle fibers from the FDB was assessed across three independent biological replicates (*n* = 3). FDB muscles were extracted from the hind legs of three mice, with each pair of FDB muscles treated as a biological replicate (*n* = 1). For each experimental “*n*”, 50 isolated FDB fibers were analyzed per condition, resulting in a total of 150 isolated FDB fibers evaluated per condition across all replicates.

### 2.12. Determination of Reactive Oxygen Species (ROS)

Myotubes and single-isolated FDB fibers were incubated with rCCL5 at 200 ng/mL for 48 h. ROS levels were assessed in single-isolated FDB fibers using the CM-DCF-DA probe (Invitrogen catalog#C6827, Waltham, MA, USA) for 30 min. Subsequently, fibers were visualized using the Motic BA310 epifluorescence microscope (Motic, Hong Kong), and the images were analyzed with the ImageJ software. Quantification was performed using the mean fluorescence intensity normalized by the area of the muscle fibers. The ROS determination in isolated muscle fibers from the FDB was assessed across three independent biological replicates (*n* = 3). FDB muscles were extracted from the hind legs of three mice, with each pair of FDB muscles treated as a biological replicate (*n* = 1). For each experimental “*n*”, 50 isolated FDB fibers were analyzed per condition, resulting in a total of 150 isolated FDB fibers evaluated per condition across all replicates.

### 2.13. Determination of Carbonylated Protein Levels

The detection of carbonylated proteins in myotubes was assessed by immunoblot using the OxyBlot assay (OxyBlot Protein Oxidation Detection Kit, Millipore, S7150). Briefly, 40 μg of total proteins were subjected to SDS-PAGE and transferred to PVDF membranes, which were incubated with the antibodies in the kit. Images were captured using the Fotodyne FOTO/Analyst Luminary Workstation Systems (Fisher Scientific, Waltham, MA, USA), and band intensity quantification was performed using densitometric analysis with ImageJ software (NIH, Bethesda, MD, USA).

### 2.14. Mitochondrial ROS (mtROS) and Mitochondrial Membrane Potential Analysis

The mtROS levels and mitochondrial membrane potential were measured using flow cytometry. The myotubes were treated with 200 ng/mL of rCCL5 for 72 h, followed by washes with DMEM. Then, the myotubes were incubated with 10 μM of MitoSOX Red probe (Invitrogen catalog#M36008, Waltham, MA, USA) or 400 nM of TMRE in DMEM 4% horse serum for 30 min. Myotubes were washed, trypsinized, and centrifuged at 1000× *g* for 10 min. The pellet was resuspended in PEB buffer (PBS-EDTA 2 mM-BSA 0.5%). Finally, the myotubes were analyzed by flow cytometry using FACSymphony^TM^ A1 equipment (BD Biosciences, San Jose, CA, USA). The mean fluorescence intensity (MFI) was determined in the samples after acquiring 500,000 total events. The MFI was normalized by the number of myotubes, quantified for each condition, and compared to the vehicle group.

### 2.15. Transmission Electron Microscopy

The myotubes were treated with 200 ng/mL of rCCL5. After 72 h, the myotubes were fixed in a glutaraldehyde 4% solution. Three washes with sodium cacodylate buffer 0.1 M were made before 2 h of osmium tetroxide 2% staining. Also, a second staining was done for 2 h with uranyl acetate and posterior washes with 1% cacodylate buffer. Dehydration with acetone gradients was made, and myotubes were embedded in Epon [18]. Sections measuring 80 nm were mounted on electron microscopy grids for examination using a transmission electron microscope (Philips, Tecnai 12 at 80 kV). For each independent experiment, the cell mitochondria of at least three fields in four myotubes counted 6–8 mitochondria per field. The mitochondrial density was calculated by the mitochondrial percentage of the total area, and the mitochondrial size was determined in nm^2^. The width/length ratio was calculated to obtain mitochondrial circularity. The mitochondrial cristae number was normalized by the mitochondrial size. Finally, the number of mitophagosome-like structures was counted manually. An expert made all quantifications blindly at 20,000× magnification.

### 2.16. Mitochondrial Oxygen Consumption

The C_2_C_12_ myoblasts were plated on seahorse plates and differentiated over 5 days into myotubes. Subsequently, they were treated with rCCL5 200 ng/mL for 48 h. The treatment medium was changed to Seahorse assay medium by supplementing with 5 mM glucose and 10 mM sodium pyruvate, pH 7.4. Oligomycin, FCCP, and Rotenone/Antimycin were injected sequentially during the trial. Oxygen consumption (OCR) was measured 3 times after each injection in Seahorse XF24 from Agilent (Seahorse Bioscience, North Billerica, MA, USA). OCR measurements were normalized by total protein content in each well. 

### 2.17. Statistics

Statistical data analysis was conducted using Prism 9.0 analysis software (GraphPad Software, San Diego, CA, USA). The normality of the data was determined through the Kolmogorov–Smirnov test. Data were analyzed with a *t*-test or one-way ANOVA, followed by a Tukey post-hoc test. Differences were considered significant when *p* < 0.05.

## 3. Results

### 3.1. CCL5 Overexpression in Skeletal Muscle Decreased Fiber Diameter and Sarcomeric Protein Levels

To assay the direct impact of CCL5 on skeletal muscle, we conducted an overexpression study in the TA muscle using electroporation of a plasmid carrying the coding sequence for the mouse *ccl5* gene (oeCCL5) or a control plasmid (Mock). To confirm the overexpression of CCL5, we evaluated *ccl5* gene expression by RT-qPCR analysis in the TA muscle at 7 days post-electroporation. The results revealed an upregulation of *ccl5* mRNA at 7 days (4.0-fold increase) (Supplementary Figure S1A) accompanied by an increase in CCL5 protein levels (15.0-fold increase) (Supplementary Figure S1B,C). Since the plasmids used to deliver the *ccl5* gene or Mock are bicistronic and express GFP independently, we performed the immunofluorescent detection of the GFP-positive area on transverse sections of the TA muscles at 7 and 21 days post-electroporation (Supplementary Figure S2A,D). The results show that GFP was expressed similarly in the Mock and oeCCL5 groups (Supplementary Figure S2A,D). This result was further confirmed by measuring *gfp* gene expression, indicating no changes in mRNA levels in the oeCCL5 and Mock groups (Supplementary Figure S2C,F). Therefore, these results together demonstrate the effective overexpression of *ccl5* through electroporation.

**Figure 1:**
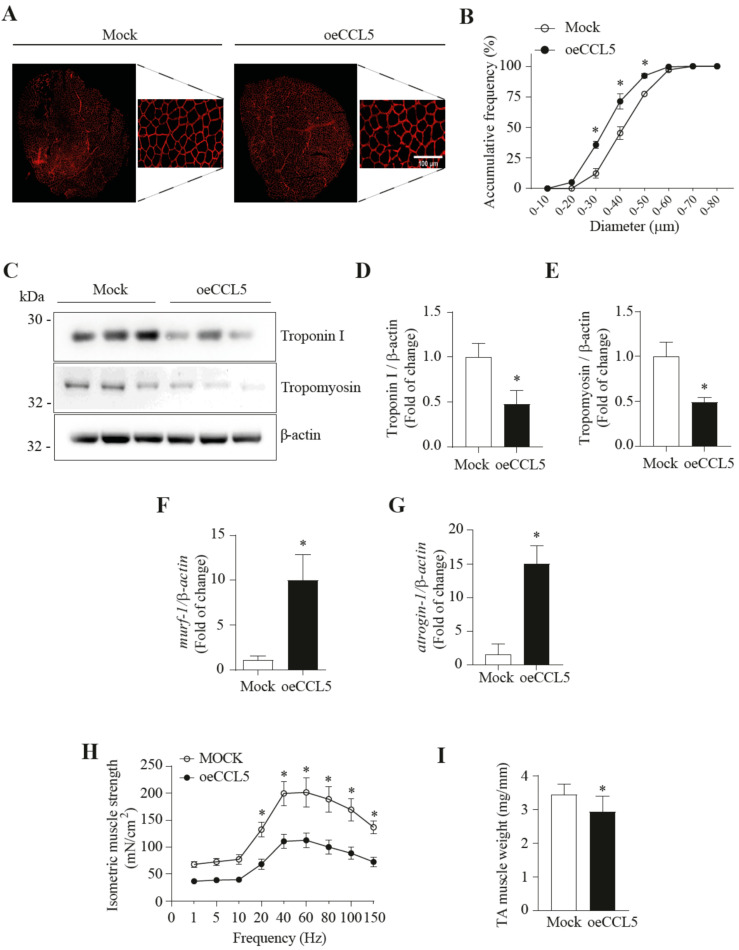
**Overexpression of CCL5 produces sarcopenia in TA.** Male C57BL6 mice were electroporated with control plasmid (Mock) and CCL5 overexpression plasmid (oeCCL5) in the tibialis anterior (TA) muscle for 21 days. (**A**) Immunofluorescence anti-laminin in TA sections of 10 µm thickness, the left image corresponds to 10X magnification, and the right image corresponds to digital magnification; the scale bar is 100 µm. (**B**) A cumulative frequency graph of fiber diameters is expressed in % corresponding to a range of diameters. (**C**) Protein levels of Troponin I and tropomyosin, using β-actin as a loading control. (**D**,**E**) densitometric analysis of Troponin I and Tropomyosin protein levels, respectively. (**F**,**G**) mRNA levels of murf-1 and atrogin-1, respectively, using β-actin as housekeeping. (**H**) Measurement of isometric force in TA, using 1–150 Hz frequencies and normalized for tibia length. (**I**) Determination of the weight of TA: the weights were normalized using the corresponding tibia of each animal. The results are expressed as the mean ± SD (*n* = 4, ANOVA two-way post hoc Bonferroni; *t*-test * *p* < 0.05 vs. Vehicle).

To evaluate possible muscular alterations related to sarcopenia due to the overexpression of CCL5, we first assessed the diameter of muscle fibers in the TA muscles after 7 and 21 days of electroporation. Specifically, we delimited muscle fiber using an anti-laminin antibody, which was detected by indirect immunofluorescence (Figure 1A and Supplementary Figure S3A). Our findings reveal that CCL5 overexpression (oeCCL5) in TA muscles showed no changes in the fiber diameter at 7 days post-electroporation (Supplementary Figure S3A,B). However, 21 days after electroporation, CCL5 overexpression decreased fiber diameter compared to the Mock group (Figure 1A,B). A more detailed analysis on day 21 indicated a higher abundance of fibers smaller than 40 μm (range from 0 to 40 μm) in oeCCL5 compared to the Mock group (71.3 ± 6.3% vs. 45.3 ± 5.2%) (Figure 1B). 

A possible cause of the decrease in fiber diameter is the decrease in the content of sarcomeric proteins. Using western blot analysis, we evaluated the effect of CCL5 overexpression in TA muscles on the protein levels of sarcomeric proteins such as troponin I and tropomyosin at 21 days post-electroporation (Figure 1C). We observed that CCL5 overexpression (oeCCL5) reduced troponin I and tropomyosin protein levels by approximately 48% (0.48 ± 0.15- vs. 1.00 ± 0.15-fold) (Figure 1D) and 49% (0.49 ± 0.05- vs. 1.00 ± 0.16-fold) (Figure 1E) compared to the Mock group. 

These findings indicate that the CCL5 overexpression in the TA muscle leads to a consistent reduction in fiber diameter at 21 days after electroporation and decreases the content of sarcomeric proteins, thereby impacting the structural integrity of the TA muscle.

### 3.2. Overexpression of CCL5 in Skeletal Muscle Increased MuRF-1 and Atrogin-1 Gene Expression

One of the mechanisms of protein degradation implicated in sarcopenia is the ubiquitin–proteasome system (UPS). MuRF-1 and atrogin-1 are two E3 ligases increased in sarcopenic conditions and responsible for ubiquitinating specific sarcomeric proteins targeted for degradation by the proteasome. In this context, we investigated whether the CCL5 overexpression in the TA muscle could affect *murf-1* and *atrogin-1* gene expression. Our findings reveal that the oeCCL5 group exhibits increased gene expression of murf-1 (9.97 ± 2.90- vs. 1.00 ± 0.48-fold) (Figure 1F) and *atrogin-1* (14.97 ± 2.72- vs. 1.00 ± 1.61-fold) (Figure 1G) compared with the Mock group. This finding was consistent with previous results from our laboratory, where we observed that rCCL5 induced proteasome activity [17].

This result suggests that CCL5 overexpression contributes to the upregulation of UPS components in TA muscle.

### 3.3. Overexpression of CCL5 in Skeletal Muscle Leads to Sarcopenia

Once we observed the decreased fiber size and molecular alterations classically observed in sarcopenia, we evaluated two parameters associated with sarcopenia in ccl5-overexpressing TA muscles, such as muscle strength and mass. The results reveal that TA muscles overexpress CCL5 and decrease muscle strength 21 days post-electroporation, indicating a lower muscle force from 20 Hz to 150 Hz (Figure 1H). To confirm that the electroporation methodology does not affect the basal strength of the mice, we incorporated a group that was not subjected to electroporation (No electr), and we observed a similar response to the Mock group (Supplementary Figure S3C).

In parallel, we evaluated muscle mass 21 days after electroporation, observing a decrease in muscle mass of 15% in comparison with the Mock group (Figure 1I). 

Upon confirming that the overexpression of CCL5 can induce sarcopenia in skeletal muscle, we analyzed the *ccr5* (Supplementary Figure S4A,B) and *ccr1* (Supplementary Figure S4C,D) gene expression. We only observed increased *ccr5* expression at 21 days post-electroporation in oeCCL5 compared with Mock. No changes between Mock and oeCCL5 were visualized in the *ccr1* and *ccr5* expression on the other days.

These findings indicate that the CCL5 overexpression in the TA muscle leads to sarcopenia 21 days post-electroporation, decreasing muscle mass and muscle strength.

### 3.4. CCL5, Through the CCR5 Receptor, Produces a Sarcopenic-like Phenotype in Muscle Cells

**Figure 2:**
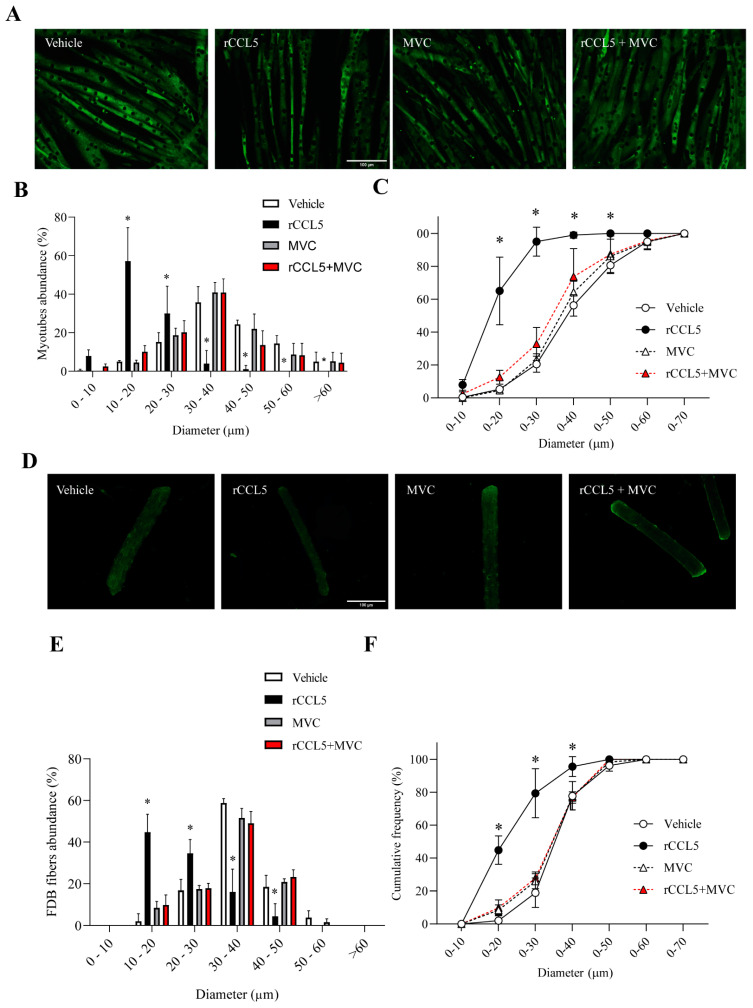
**CCL5 via the CCR5 receptor decreases the diameter of muscle cells.** C_2_C_12_ myoblast cell lines, differentiated for 5 days into myotubes and muscle fibers isolated from FDB, were pretreated with 10 µM of the CCR5 inhibitor (Maraviroc, MVC) for 1 h and subsequently treated with 200 ng/mL of rCCL5 for 72 h. (**A**) Surface delineation of C_2_C_12_ myotubes by immunofluorescence against Cav-3, magnification corresponding to 20X, and the scale bar corresponds to 100 µm. (**B**) Quantification of the diameter of C_2_C_12_ myotubes, using an abundance graph expressed in % vs. range of myotube diameters. (**C**) Graph of cumulative frequency expressed in % of C_2_C_12_ myotubes vs. diameter. (**D**) Delineation of muscle fibers isolated from FDB by immunofluorescence anti-Cav-3, magnification corresponding to 20X, and scale bar corresponding to 100 µm. (**E**) Quantification of the diameter of FDB muscle fibers by abundance plot expressed in % relative to muscle-fiber size ranges (µm). (**F**) FDB muscle-fiber diameter quantification plotted as cumulative frequency (%) versus diameter. The results are expressed as the mean ± SD (*n* = 3). Eighty myotubes and 50 fibers were analyzed per condition for each “*n*” (a total of 240 myotubes and 150 fibers for each condition). ANOVA two-way post hoc Bonferroni * *p* < 0.05 vs. Vehicle).

We investigated whether cultured muscle cells incubated with recombinant CCL5 (rCCL5) exhibit a sarcopenic-like phenotype. For this, we first determined the concentration of rCCL5 that affects cell viability through the MTT assay (Supplementary Figure S5A) and produced an alteration in fiber diameter from FDB (Supplementary Figure S5B), determining that the concentration of 200 ng/mL did not affect cell viability and produced a sarcopenic-like phenotype. Then, we evaluated the involvement of CCR1 and CCR5 receptors in the sarcopenic-like phenotype produced by rCCL5. We first evaluated the presence of CCR1 and CCR5 receptors in FDB muscle fibers and C_2_C_12_ myotubes (Supplementary Figure S6A,B), showing that both receptors are expressed in muscle fibers and myotubes. Subsequently, we evaluated the participation of CCR1 and CCR5 receptors in the diameter of isolated FDB muscle fibers and C_2_C_12_ myotubes. Initially, we assessed the impact of rCCL5 on the diameter of C_2_C_12_ myotubes and single-isolated FDB fibers. We evaluated the participation of the CCR1 receptor through the use of the inhibitor J113863 in the decrease in the diameter of muscle fibers isolated from FDB and C_2_C_12_ myotubes (Supplementary Figure S6C,D). Indirect immunofluorescence against caveolin 3 (Cav-3) was performed to outline the myotube surface, and no changes were observed in the decrease in diameter in both cell types mediated by rCCL5, indicating that this receptor would not be participating in this sarcopenic-like phenotype. Using the same technique, we also assayed the participation of the CCR5 receptor by incubation with the CCR5 receptor-specific antagonist Maraviroc (MVC) (Figure 2A,D). In Figure 2B, we observed a notorious leftward shift in the curve of diameter vs. abundance when C_2_C_12_ myotubes were incubated with rCCL5. The same figure shows the highest abundance of myotubes corresponds to the diameter range between 30 and 40 µm in the vehicle group (35.76 ± 8.15%), while in the rCCL5 group, it is found in minor diameters, specifically in the range between 10 and 20 µm (57.10 ± 17.45%). This result indicates that CCL5 decreased the myotube diameter. In parallel, we observed that using MVC completely prevented this decrease in the diameter of myotubes treated with rCCL5, restoring the curve to that observed in the vehicle (Figure 2B).

This finding is reinforced by the analysis of the accumulated frequency, which indicates that nearly 100% (94.97 ± 8.72%) of myotubes in the rCCL5 group had a diameter thinner than 30 µm. In contrast, approximately 20% (20.47 ± 4.86%) were observed in the vehicle group in the same diameter range (Figure 2C). MVC reinstated the proportion of myotubes with diameters thinner than 30 µm in the rCCL5 group, resembling the values seen in the vehicle group.

These results align with observations in single muscle fibers isolated from FDBs incubated with rCCL5 for 72 h (Figure 2D), which show an evident leftward shift in the curve of diameter vs. abundance (Figure 2E). Furthermore, Figure 2F demonstrates that the rCCL5 group accumulated approximately 75% (74.97 ± 7.60%) of fibers with a diameter thinner than 30 µm, compared to only 20% (18.93 ± 8.89%) in the vehicle group. Using the MVC inhibitor completely prevented the decrease in the diameter of muscle fibers isolated from FDB (Figure 2E,F).

Together, these results demonstrated that CCR5, but not the CCR1 receptor, is mediating the alterations in myotubes and isolated fiber diameters induced by CCL5.

**Figure 3:**
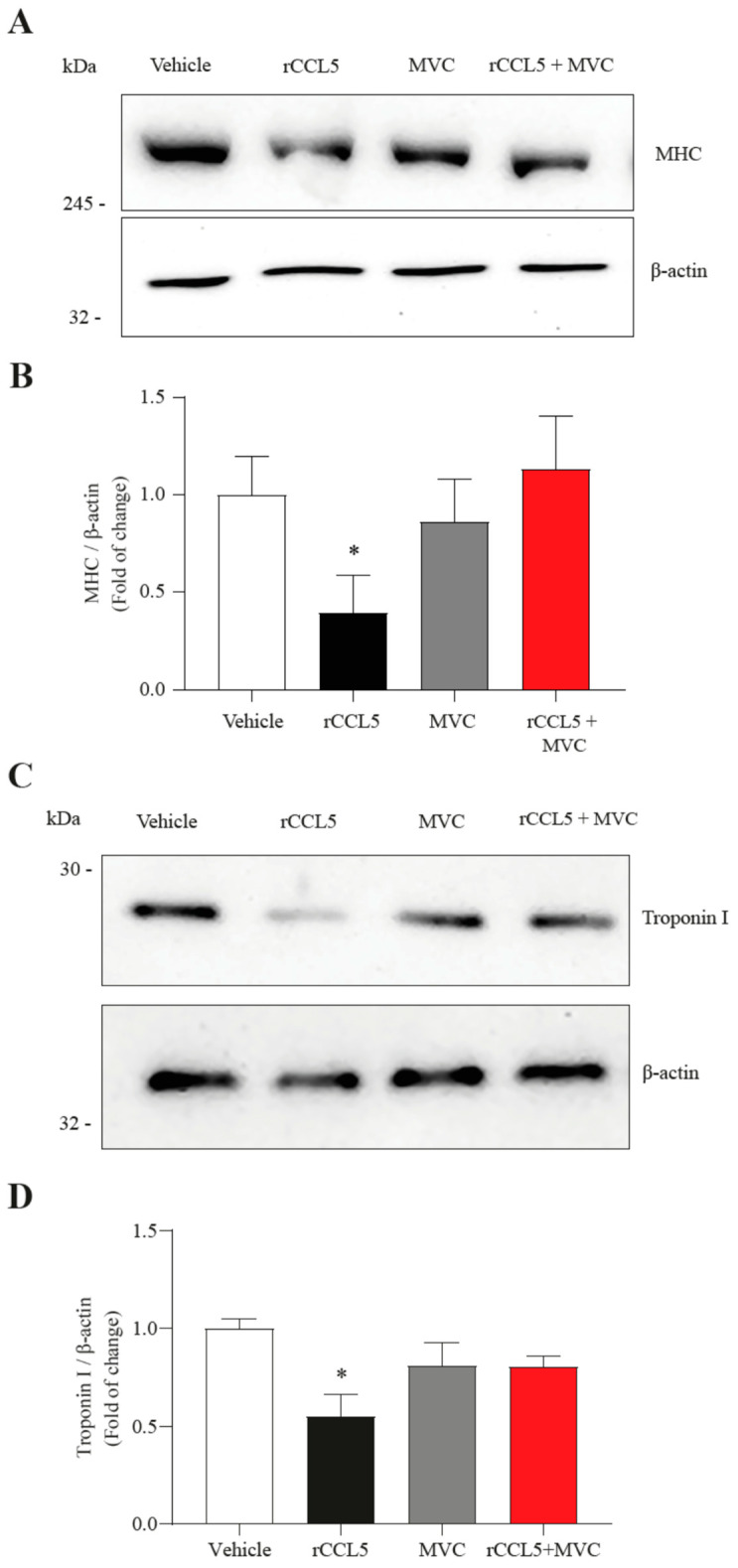
**CCL5, through the CCR5 receptor, decreases the protein levels of sarcomere components in muscle cells.** C_2_C_12_ myoblast cell lines differentiated for 5 days into myotubes were preincubated with 10 µM of the CCR5 antagonist (Maraviroc, MVC) for 1 h and subsequently incubated with 200 ng/mL of rCCL5 for 72 h. (**A**,**C**) Protein levels of sarcomere component proteins, MHC, and troponin I, respectively, using β-actin as a loading control. (**B**,**D**) Densitometric analysis of the protein levels of MHC and troponin I, respectively. The results are expressed as the mean ± SD (*n* = 3, one-way ANOVA post hoc Bonferroni * *p* < 0.05 vs. vehicle).

To confirm the sarcopenic-like phenotype observed in muscle cells, we assessed whether treatment with rCCL5 in C_2_C_12_ myotubes would impact the protein levels of MHC (Figure 3A) and troponin I (Figure 3C) by Western blot analysis. We observed that rCCL5 decreased MHC (Figure 3B) and troponin I (Figure 3D) protein levels by nearly 75% (1.00 ± 0.01- vs. 0.25 ± 0.15-fold) and 40% (1.00 ± 0.1- vs. 0.63 ± 0.13-fold), respectively, compared to vehicle-treated C_2_C_12_ myotubes. Moreover, we evaluated the impact of MVC on the effect of CCL5 over the protein levels of MHC and troponin I (Figure 3). The results show that MVC completely prevented the rCCL5-induced decrease of MHC protein levels (rCCL5: 0.39 ± 0.19-fold; rCCL5+MVC: 1.13 ± 0.27-fold) (Figure 3A,B). We observed the same behavior with troponin I protein levels (rCCL5: 0.55 ± 0.12-fold; rCCL5+MVC: 0.81 ± 0.05-fold) (Figure 3C,D), resembling levels observed in the vehicle group.

These findings indicate that CCL5, through the CCR5 receptor, induces a sarcopenic-like phenotype in C_2_C_12_ myotubes and single-isolated FDB muscle fibers.

### 3.5. CCL5 Increases UPS Components and Attenuates Protein Synthesis in Myotubes and Single-Isolated Muscle Fibers

**Figure 4:**
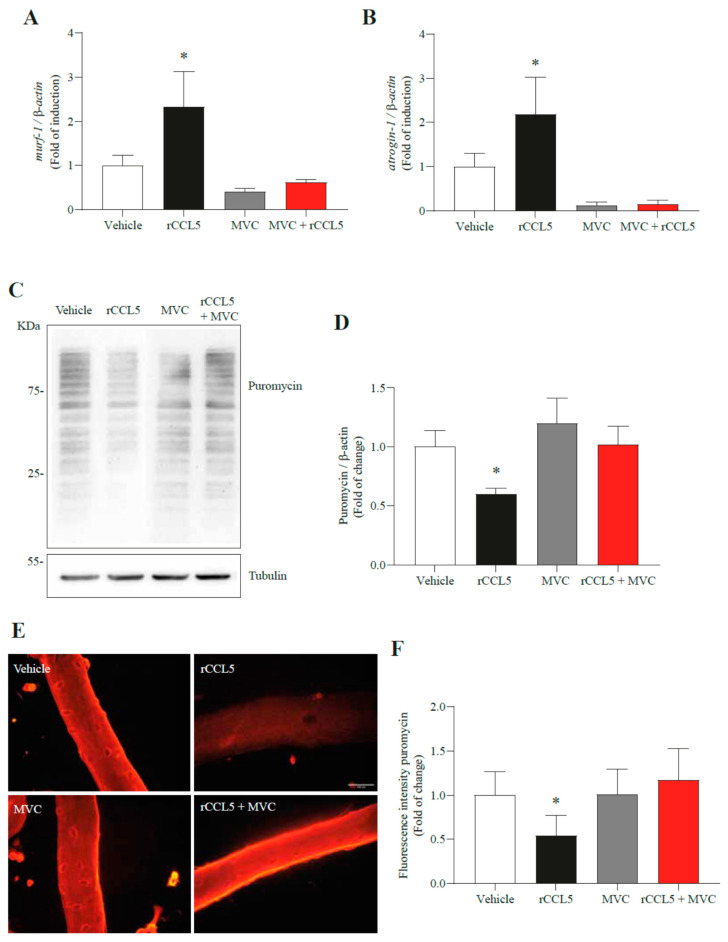
**CCL5, via the CCR5 receptor, deregulates protein synthesis and degradation processes in muscle cells.** C_2_C_12_ myoblasts were differentiated for 5 days into myotubes, and muscle fibers isolated from FDB were pretreated with 10 µM of the CCR5 inhibitor (Maraviroc, MVC) for 1 h and subsequently treated with 200 ng/mL of rCCL5 for 24 h. (**A**,**B**) mRNA levels of the E3s ligases *murf-1* and *atrogin-1* respectively, using *β-actin* as housekeeping. (**C**) Puromycin incorporation assay in C_2_C_12_ myotubes, determined through protein levels using β-actin as a loading control. (**D**) Densitometric analysis of puromycin levels in C_2_C_12_ myotubes. (**E**) Puromycin assay in FDB muscle fibers, determined through immunofluorescence with anti-puromycin antibody, magnification of the images corresponding to 40X, scale bar corresponding to 100 µm. (**F**) Quantification of puromycin incorporation through fluorescence intensity of FDB fibers. The results are expressed as the mean ± SD (*n* = 3). Fifty fibers were analyzed per condition for each “*n*” (a total of 150 fibers for each condition). One-way ANOVA post hoc Bonferroni * *p* < 0.05 vs. Vehicle).

During sarcopenia, an imbalance occurs between the synthesis processes and protein degradation in muscle cells. As mentioned above, the primary mechanism of protein degradation in sarcopenia is the UPS, considering MuRF-1 and atrogin-1 as critical elements of this mechanism. We previously showed an upregulation of *murf-1* and *atrogin-1* gene expression by CCL5 overexpression in TA muscles. Then, we determined whether CCL5 could influence *murf-1* and *atrogin-1* gene expression in C_2_C_12_ myotubes. We observed an increment in the gene expression of *murf-1* at 24 h in C_2_C_12_ myotubes treated with rCCL5 compared to those treated with a vehicle (1.00 ± 0.24- vs. 2.43 ± 0.59-fold) (Figure 4A). Conversely, it was noted that *atrogin-1* exhibited an increased gene expression at 12 h (1.00 ± 0.15- vs. 1.83 ± 0.73-fold) and 24 h (1.00 ± 0.16- vs. 1.78 ± 0.50-fold) after treatment with rCCL5 when compared to vehicle-treated C_2_C_12_ myotubes (Figure 4B). Additionally, we determined that the use of MVC completely prevented the increase of gene expression of both UPS components (Figure 4A,B).

Protein synthesis is another process susceptible to alterations during sarcopenia. To ascertain the impact of CCL5 on protein synthesis in C_2_C_12_ myotubes and single-isolated FDB muscle fibers, we employed the SunSET assay (puromycin incorporation). This assay provides insights into the rate of cellular protein synthesis, and it can be evaluated through Western blot and indirect immunofluorescence (Figure 4C,E). Through Western blot analysis, we observed that the 24 h treatment of C_2_C_12_ myotubes with rCCL5 led to approximately a 25% reduction in puromycin incorporation (Figure 4C) compared to vehicle-treated C_2_C_12_ myotubes (1.00 ± 0.01 vs. 0.74 ± 0.05-fold) (Figure 4D). This observation was further corroborated in muscle fibers isolated from FDB muscles and incubated with rCCL5 for 24 h (Figure 4E), demonstrating lower fluorescence intensity than muscle fibers treated with a vehicle (1.00 ± 0.39- vs. 0.43 ± 0.18-fold) (Figure 4F). Furthermore, we assessed whether the decrease in the rate of protein synthesis induced by CCL5 depended on the CCR5 receptor. As observed in Figure 4C,D, the reduction in the rate of protein synthesis in C_2_C_12_ myotubes produced by rCCL5 (0.6 ± 0.1-fold) was entirely prevented by antagonizing the CCR5 receptor with MVC (vehicle: 1.0 ± 0.1-; MVC: 1.2 ± 0.2-; rCCL5+MVC: 1.1 ± 0.1-fold). This result was corroborated in single-isolated FDB muscle fibers treated with rCCL5 in the absence or presence of MVC. The decreased fluorescent intensity associated with puromycin incorporation induced by rCCL5 (0.54 ± 0.23-fold) was restored by MVC to similar levels to the vehicle group (vehicle: 1.00 ± 0.01-; MVC: 1.01 ± 0.28-; rCCL5+MVC: 1.17 ± 0.36-fold) (Figure 4E,F).

These findings indicate that CCL5, through the CCR5 receptor, produces an imbalance between the processes of protein synthesis and degradation, upregulates the gene expression of E3 ligases associated with UPS, and decreases the rate of protein synthesis, contributing to the manifestation of a sarcopenia-like phenotype in muscle cells.

### 3.6. CCL5 Produces Oxidative Stress and Augmented Reactive Oxygen Species Production in Single-Isolated Muscle Fibers

**Figure 5:**
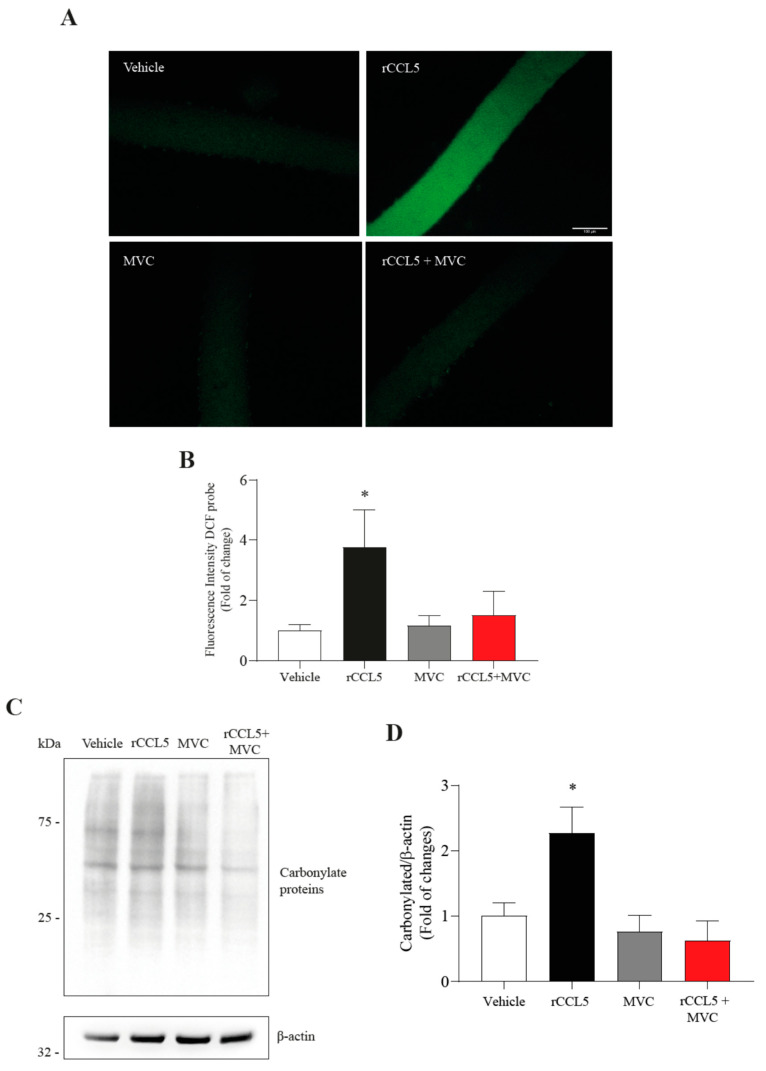
**CCL5, through the CCR5 receptor, produces oxidative stress, increasing the production of ROS in muscle cells.** C_2_C_12_ myoblast cell lines were differentiated for 5 days into myotubes, and muscle fibers isolated from FDB were pretreated with 10 µM of the CCR5 inhibitor (Maraviroc, MVC) for 1 h and subsequently treated with 200 ng/mL of rCCL5 for 48 h. (**A**) Reactive oxygen species (ROS) are detected through DCF in FDB muscle fibers. Magnification of 40X and the corresponding scale bar at 100 µm. (**B**) Quantification of ROS production through fluorescence intensity of the DCF probe. (**C**) Determination of protein levels of carbonylated proteins in C_2_C_12_ myotubes using Oxyblot, using β-actin as a loading control. (**D**) Densitometric analysis of protein carbonylation. The results are expressed as the mean ± SD (*n* = 3). Fifty fibers were analyzed per condition for each “*n*” (a total of 150 fibers for each condition). One-way ANOVA post hoc Bonferroni * *p* < 0.05 vs. Vehicle).

The increase in reactive oxygen species (ROS) is a significant factor in sarcopenia, as it can induce oxidative stress in muscle cells, leading to disruptions in protein metabolism. We assessed reactive oxygen species (ROS) levels in single-isolated FDB muscle fibers incubated with recombinant CCL5 (rCCL5) using the DCF probe (Figure 5A). As shown in Figure 5B, rCCL5 treatment resulted in an approximately fourfold increase in ROS production at 48 h post-treatment (1.00 ± 0.67 vs. 3.77 ± 1.21-fold). Additionally, we evaluated whether this increase in ROS could induce oxidative stress in C_2_C_12_ myotubes through Western blot analysis by detecting carbonylated proteins. The results revealed that rCCL5 significantly increased carbonylated proteins compared to the vehicle group (1.00 ± 0.21 vs. 3.1 ± 0.07) (Figure 5C,D), indicating the induction of oxidative stress in muscle cells. In parallel, we observed that using MVC effectively prevented the oxidative stress induced by CCL5, inhibiting ROS production in isolated FDB muscle fibers (Figure 5A) and preventing protein carbonylation in C2C12 myotubes (Figure 5C).

These findings indicate that CCL5, through the CCR5 receptor, produces oxidative stress in muscle cells.

### 3.7. CCL5 Did Not Alter the Mitochondrial Function in Skeletal Muscle Cells

Alterations in mitochondria have also been described as a mechanism that can lead to sarcopenia. Therefore, we evaluated the influence of CCL5 on the morphological parameters of the mitochondria in C_2_C_12_ myotubes treated with rCCL5 by transmission electron microscopy. Our results show that rCCL5 did not alter the morphology of the mitochondria (Supplementary Figure S7A) or their size (Supplementary Figure S7B). In addition, we observed that rCCL5 did not change the mitochondrial cristae density (Supplementary Figure S7C) or the content of mitophagosome-like structures (Supplementary Figure S7D). Additionally, we observed that rCCL5 did not alter the ATP-associated oxygen consumption rate (Supplementary Figure S7E). 

Furthermore, we evaluated mitochondrial ROS production through the detection of fluorescent probe Mitosox by flow cytometry (Supplementary Figure S8A). Our results indicate that rCCL5 treatment does not alter mitochondrial ROS production in C_2_C_12_ myotubes. Finally, we measured the effect of rCCL5 on the mitochondrial membrane potential using the TMRE probe through flow cytometry. The results show that rCCL5 does not alter the mitochondrial membrane potential (Supplementary Figure S8B). 

These results indicate that CCL5 does not affect the parameter of mitochondrial function evaluated.

## 4. Discussion

CCL5 is a new myokine whose expression is regulated by muscle contraction [15]. Despite elevated CCL5 levels having been reported in sarcopenic skeletal muscle induced by sepsis [24] and cholestatic chronic liver disease [12], its role in the generation and development of sarcopenia has been unexplored. The present study establishes that CCL5 overexpression in skeletal muscle can induce sarcopenia. In addition, we determined that cultures of myotubes or FDB-isolated fibers incubated with rCCL5 show that the CCR5 receptor mediates the sarcopenic effect.

In sarcopenia, pathological features such as decreased muscle-fiber diameter and the levels of sarcomeric proteins have been described. Our findings demonstrate that the overexpression of CCL5 in TA induces sarcopenia 21 days after electroporation, decreasing muscle mass and the ability to generate force in the TA muscle. The sarcopenia produced by CCL5 was accompanied by a reduction in muscle-fiber diameter and a decrease in sarcomeric proteins, specifically tropomyosin and troponin I. This effect was replicated with rCCL5 in cell culture, showing decreased diameter of myotubes and single-isolated FDB fibers and diminished troponin I protein content. These alterations suggest that CCL5 potentially disrupts the structure and functionality of skeletal muscle. Notably, the sarcopenic-like effect was prevented entirely when muscle cells were pre-treated with MVC, a specific CCR5 antagonist, supporting the participation of the CCR5 receptor in these sarcopenic alterations induced by CCL5.

The reduction in muscle-fiber diameter and the decline in protein levels of sarcomere components observed in diverse sarcopenia models are associated with various molecular mechanisms, including increased protein degradation through the ubiquitin–proteasome system (UPS). Our study revealed that the overexpression of CCL5 in the TA muscle and its incubation in muscle cells led to an upregulation in the mRNA levels of MuRF-1 and atrogin-1. These E3 ligases are implicated in sarcopenia, targeting the ubiquitination of sarcomere proteins such as troponin I and MHC. Other studies have observed the correlation between increased expression of MuRF-1 and atrogin-1 with decreased protein content [25]. Notably, in sarcopenia due to denervation, the elevated expression of MuRF-1 has been identified as critical in the onset of sarcopenia, as demonstrated by a protective role when this ligase is knocked out [26]. In this line of evidence, we recently showed that UPS plays a key role in decreasing the diameter of FDB-isolated fibers and reducing the protein levels of MHC and troponin I [17], consistent with our observations in this study. 

The mechanisms activated by CCL5 in skeletal muscle and downstream of the CCR5 receptor have not been elucidated. In this context and based on our results, two transcription factors, FoxO and NF-κB, appear to be possible candidates for regulating the sarcopenic actions mediated by CCL5. In a hyperlipidemia model, CCL5 modulates NF-κB activation through the CCR5 receptor in smooth muscle cells [27]. This mechanism involves the activation of PI3K, ERK1/2, and p38 signaling pathways. In addition, during liver regeneration, CCL5, via CCR5 as a critical receptor, activates FoxO3a in macrophages, inducing their differentiation into pro-inflammatory macrophages and impacting tissue regeneration [28]. Notably, FoxO [29] and NF-κB [30] are critical regulators of UPS influencing sarcopenia. These findings support the hypothesis that CCL5, via the CCR5 receptor in skeletal muscle, could regulate FoxO and NF-κB, providing a plausible explanation for the observed results in our study. Further experiments about the participation of FoxO and NF-κB in the sarcopenic-like effect mediated by CCL5 must be conducted.

Complementary to the activation of proteostasis, our results also reveal that CCL5 reduces the protein synthesis in skeletal muscle cells, measured through the puromycin incorporation assay. Protein synthesis, a process susceptible to oxidative stress in sarcopenia, experiences decreased mRNA translation during the initiation stage in the presence of ROS. This effect is attributed to the reduction in phosphorylation of downstream targets of mTOR, p70S6K, and 4EBP-1 [31,32], which are all crucial in protein synthesis. This observation aligns with our results in the present study, which show that CCL5, through the CCR5 receptor, produces oxidative stress in muscle cells reported through the increased carbonylation protein and augmented ROS production in single-isolated FDB muscle fibers. These findings suggest that oxidative stress could be pivotal in diminishing the rate of protein synthesis observed in the present study.

In the same way as CCL5, other myokines have been shown to activate molecular mechanisms involved in sarcopenia. It has been described that myostatin and IL-6 can increase UPS components such as MuRF-1 and atrogin-1, decreasing the content of sarcomeric proteins and muscle mass [33,34]. On the other hand, it has also been reported that myostatin can increase the production of ROS, producing oxidative stress in muscle cells through the activation of NF-κB [35]. In this context, IL-6 has been associated with inflammatory processes in skeletal muscle, increasing its expression under oxidative stress [36]. Furthermore, it has been described that myostatin can decrease key pathways for myogenic differentiation, hypertrophy, and protein synthesis, such as Akt/TORC1/p70S6K [37]. On the contrary, IL-6 is a myokine that is increased in patients who present sarcopenia due to aging or secondary to pathology, and it is used as an indicator of the severity of sarcopenia [38]. The increase in IL-6 after physical exercise has a beneficial role in skeletal muscle through the increase in muscle mass and glucose uptake, improving the metabolic state of skeletal muscle [39,40]. However, it has not yet been described whether CCL5 can alter myogenic processes or whether, in other contexts, it can be beneficial for the muscle.

Regarding ROS production in skeletal muscle, three primary sources can be responsible for its increased levels: mitochondria, NADPH oxidase (NOX), and, to a lesser extent, xanthine oxidase [41]. In sarcopenia, mitochondrial dysfunction [42,43] and increased NOX activity [35,44,45] have been linked to oxidative stress, resulting in elevated activation of the mechanisms involved in protein degradation, like UPS and autophagy. Previous reports indicate that CCL5, via CCR5, can modulate NOX activity, heightening ROS levels in endothelial cells [46]. These findings explain the observed rise in ROS in single-isolated FDB fibers in our study. Considering that the results obtained in this work regarding mitochondrial functionality have shown that CCL5 did not affect the mitochondrial size or morphology and did not elevate mitochondrial ROS, it suggests that NOX can mediate the increased ROS observed in our study. Further studies using strategies to abolish NOX activity or expression of some critical subunits must be conducted to assess the role of NOX in the sarcopenic-like effect mediated by CCL5.

We do not observe changes induced by CCL5 in mitochondrial size, morphology, or phagosome-like structure. However, future studies to analyze the influence of CCL5 on some processes that regulate mitochondrial homeostasis—specifically, alterations in mitochondrial biogenesis, dynamics, or mitophagy, which have been previously shown to be altered in several conditions of sarcopenia [47,48]—could be conducted. Interestingly, our group has described alterations in these mitochondrial processes during sarcopenia induced by chronic cholestatic liver disease. Interestingly, we also showed increased muscular CCL5 expression under this pathological condition, sustaining its possible role in this sarcopenic condition [12]. It is also known that CCL5 is a myokine that decreases its expression in response to muscle contraction [15], which could correlate with the protective effect of exercise on mitochondrial alterations in muscle [49,50,51]. Therefore, future investigations must be performed to evaluate these parameters in response to CCL5.

In summary, our work establishes, for the first time, that CCL5 is a myokine that induces sarcopenia in vivo. In addition, CCL5 induces a sarcopenic-like effect mediated by the CCR5 receptor in single-isolated fibers and muscle cells. This sarcopenic effect is evidenced by decreased fiber diameter and sarcomeric protein levels, as well as reduced protein synthesis, elevated gene expression of UPS components, and oxidative stress. 

## Data Availability

The original contributions presented in this study are included in the article/Supplementary Materials. Further inquiries can be directed to the corresponding authors.

## Supplementary Materials

The following supporting information can be downloaded at https://www.mdpi.com/article/10.3390/antiox14010084/s1, Supplementary Figure S1. CCL5 overexpression in TA muscle. TA muscles from C57BL/6 male mice were electroporated with a control plasmid (Mock) or a plasmid overexpressing CCL5 (oeCCL5). TA muscles were removed at 7 days post-electroporation. (A) The ccl5 gene expression was detected for RT-qPCR using β-actin as a housekeeping gene. (B) Protein levels of CCL5 using β-actin as a loading control, detected by immunoblot. (C) Densitometric analysis of B. Mean ± SD for each group (*n* = 3 mice per group), *t*-test, * *p* < 0.05 vs. Mock. Supplementary Figure S2. Electroporation efficiency in TA muscle. TA muscles from C57BL/6 male mice were electroporated with a control plasmid (Mock) or a plasmid overexpressing CCL5 (oeCCL5). TA muscles were removed 7- and 21-days post-electroporation and cryosections were obtained (10 μm). (A,D) GFP was detected in electroporated TA muscles through immunofluorescent detection with GFP antibodies. (B,E) Quantification of the positive area for GFP (%), which was normalized to the total area of electroporated TA muscles. (C,F) The gfp gene expression was detected in samples of electroporated TA muscles at 7- and 21-days post-electroporation through RT-qPCR using β-actin as a housekeeping gene. Mean ± SD for each group (*n* = 3 mice per group), *t*-test, * *p* < 0.05 vs. Mock. Supplementary Figure S3. Overexpression of CCL5/RANTES in TA does not produce sarcopenia at 7 days post electroporation. TA muscles from C57BL/6 male mice were electroporated with a control plasmid (Mock) or a plasmid overexpressing CCL5 (oeCCL5). (A) TA muscles were removed 7 days post-electroporation, and cryosections were obtained (10 μm). (B) A cumulative frequency graph of fiber diameters is expressed in % corresponding to a range of diameters. (C) Measurement of isometric force in TA, using 1–150 Hz frequencies and normalized for tibia length 21 days post-electroporation in no electroporated (No electr), Mock, and oeCCL5. Mean ± SD for each group (*n* = 3-4 mice per group), *t*-test, * *p* < 0.05 vs. Mock. # *p* < 0.05 vs. No electr. Supplementary Figure S4. CCR1 and CCR5 expression under overexpression of CCL5/RANTES in TA muscles. TA muscles from C57BL/6 male mice were electroporated with a control plasmid (Mock) or a plasmid overexpressing CCL5 (oeCCL5). No electroporated muscles are also shown (No electr). CCR5 (A,B) and CCR1 (C,D) gene expression was detected in samples of TA muscles at 7- and 21-days post-electroporation through RT-qPCR using β-actin as a housekeeping gene. Mean ± SD for each group (*n* = 3–4 mice per group), *t*-test, * *p* < 0.05 vs. No electr. # *p* < 0.05 vs. Mock. Supplementary Figure S5. Dose-dependent effect of CCL5/RANTES in muscle cells. C2C12 myoblast cell line, differentiated for 5 days into myotubes and muscle fibers isolated from FDB, incubated with increasing concentrations of CCL5/RANTES (0 up to 400 ng/mL) for 72 h. (A) Cell viability was evaluated through MTT assay. (B) Delineation of muscle fibers isolated from FDB by immunofluorescence anti-Cav-3, magnification corresponding to 20X, and scale bar corresponding to 100 µm. Quantification of the diameter of FDB muscle fibers by abundance plot expressed in % relative to muscle fiber size ranges (µm). The results are expressed as the mean ± SD (*n* = 3). 50 fibers were analyzed per condition for each “*n*” (a total of 150 fibers for each condition). Supplementary Figure S6. Presence of CCR1 and CCR5 receptors in muscle cells and participation of the CCR1 receptor in the decrease in the diameter of muscle cells treated with rCCL5. C2C12 myoblast cell line, differentiated for 5 days into myotubes and muscle fibers isolated from FDB, were pretreated with 10 µM of the CCR1 inhibitor for 1 h and subsequently treated with 200 ng/mL of rCCL5 for 72 h. (A) Presence of CCR1 and CCR5 receptors in C2C12 myotubes during the differentiation process into myotubes. (B) Presence of CCR1 and CCR5 receptors in isolated muscle fibers of FDB. (C,D) Surface delineation of C2C12 myotubes by immunofluorescence against Cav-3, magnification corresponding to 20 X, and the scale bar corresponds to 100 µm. (C) Quantification of the diameter of C2C12 myotubes, using an abundance graph expressed in % vs. range of myotube diameters and graph of cumulative frequency expressed in % of C2C12 myotubes vs. diameter. (D) Quantification of the diameter of FDB muscle fibers by abundance plot expressed in % relative to muscle fiber size ranges (µm). and diameter quantification plotted as cumulative frequency (%) versus diameter. The results are expressed as the mean ± SD (*n* = 3). 80 myotubes and 50 fibers were analyzed per condition for each “*n*” (a total of 240 myotubes and 150 fibers for each condition). ANOVA two-way post hoc Bonferroni * *p* < 0.05 vs. Vehicle. Supplementary Figure S7. CCL5 does not alter the structure of mitochondria, nor does ATP-linked OCR. C2C12 myotubes were incubated with recombinant CCL5 (rCCL5) at 200 ng/mL for 72 h. Representative mitochondrial images. For each independent experiment, mitochondria of at least three fields in four myotubes were counted as 6–8 mitochondria per field (A) Mitochondrial circularity was determined as width/lengthy ratio, (B) Mitochondrial size in nm2, (C) the number of mitochondrial cristae was normalized by mitochondria size after incubation with rCCL5. (D) Detection of mitophagosome-like structures expressed in %. (E) ATP-linked OCR (pmol/min) was determined by OCR analysis using Seahorse. The results are expressed as the mean ± SD (*n* = 3 *t*-test). Supplementary Figure S8. CCL5 does not alter mitochondrial functionality. C2C12 myotubes were incubated with recombinant CCL5 (rCCL5) at 200 ng/mL for 72 h. Myotubes were incubated with (A) 10 μM of MitoSOX and (B) 400 nM TMRE probe for the detection of mitochondrial ROS and mitochondrial membrane potential for flow cytometry analysis. Representative flow cytometry dot plot analysis is used to identify mean fluorescence intensity (MFI) for each condition. Analysis of the MFI normalized by the number of cells (n°cells) in myotubes incubated with rCCL5. Values are expressed as the mean of MFI/n°cells ± SD (*n* = 3 independent experiments).
