# Supplementary material for: CCL5 Induces a Sarcopenic-like Phenotype via the CCR5 Receptor
Source: Antioxidants (Basel). 2025 Jan 13;14(1):84. doi: 10.3390/antiox14010084 (PMC11760477; doi:10.3390/antiox14010084)
Supplement: Supplementary file 1 [file antioxidants-14-00084-s001.zip › antioxidants-3360435-supplementary.pdf]

Supplementary Figures

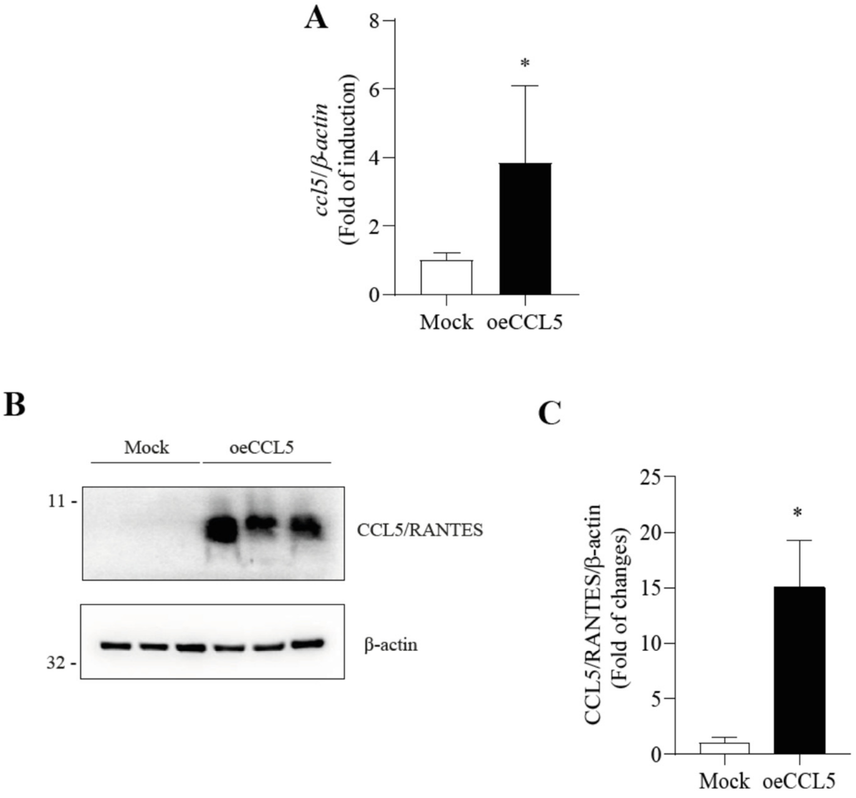

Supplementary Figure 1

Supplementary Figure 1. CCL5 overexpression in TA muscle. TA muscles from C57BL/6 male mice were electroporated with a control plasmid (Mock) or a plasmid overexpressing CCL5 (oeCCL5). TA muscles were removed at 7 days post-electroporation. **(A)** The ccl5 gene expression was detected for RT-qPCR using β-actin as a housekeeping gene. **(B)** Protein levels of CCL5 using β-actin as a loading control, detected by immunoblot. **(C)** Densitometric analysis of B. Mean ± SD for each group (n=3 mice per group), t-test, *p<0.05vs. Mock.

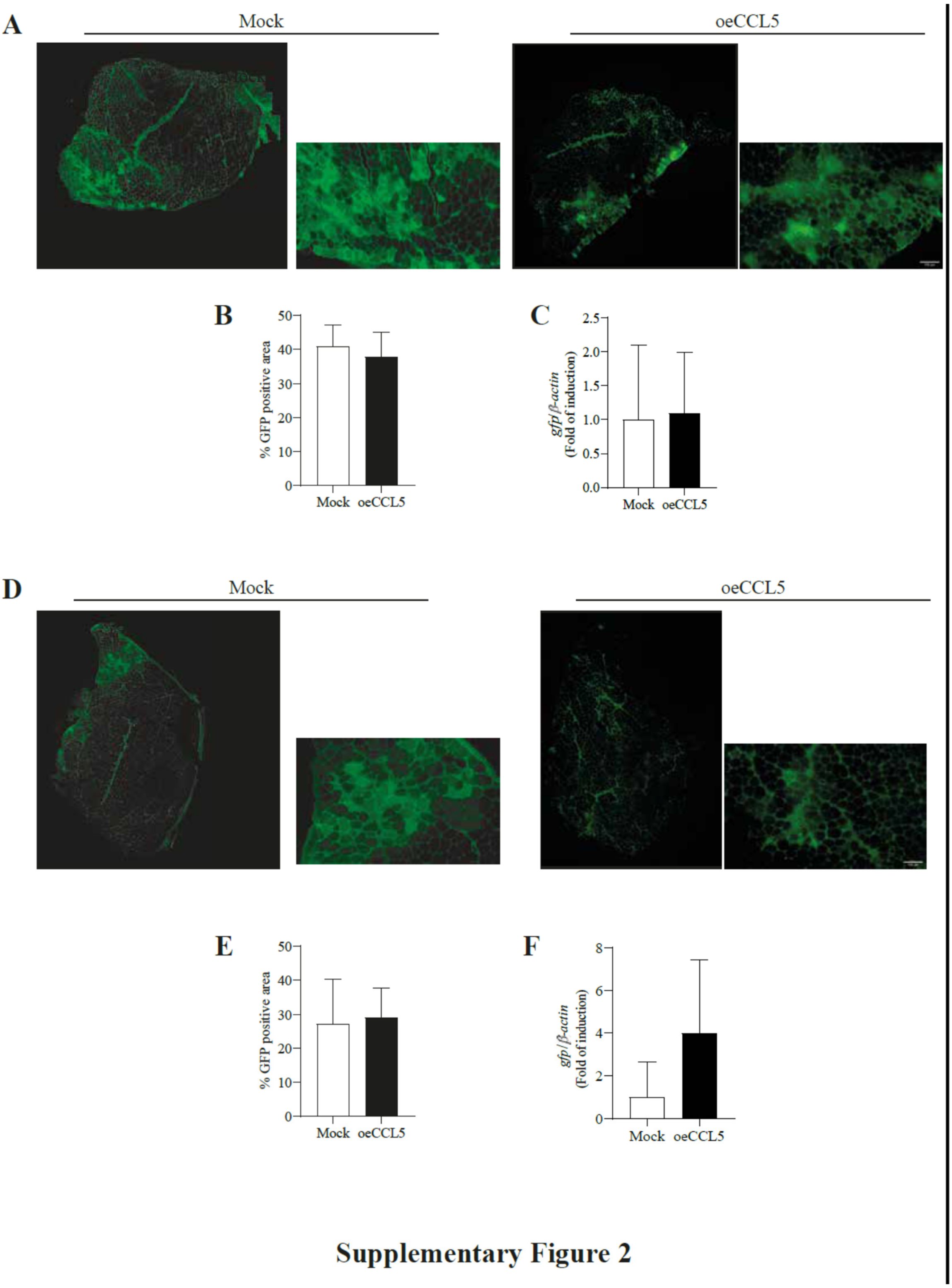

Supplementary Figure 2

Supplementary Figure 2. Electroporation efficiency in TA muscle. TA muscles from C57BL/6 male mice were electroporated with a control plasmid (Mock) or a plasmid overexpressing CCL5 (oeCCL5). TA muscles were removed 7- and 21-days post-electroporation and cryosections were obtained (10 μm). **(A, D)** GFP was detected in electroporated TA muscles through immunofluorescent detection with GFP antibodies. **(B, E)** Quantification of the positive area for GFP (%), which was normalized to the total area of electroporated TA muscles. **(C, F)** The gfp gene expression was detected in samples of electroporated TA muscles at 7- and 21-days post-electroporation through RT-qPCR using β-actin as a housekeeping gene. Mean ± SD for each group (n=3 mice per group), t-test, *p<0.05vs. Mock.

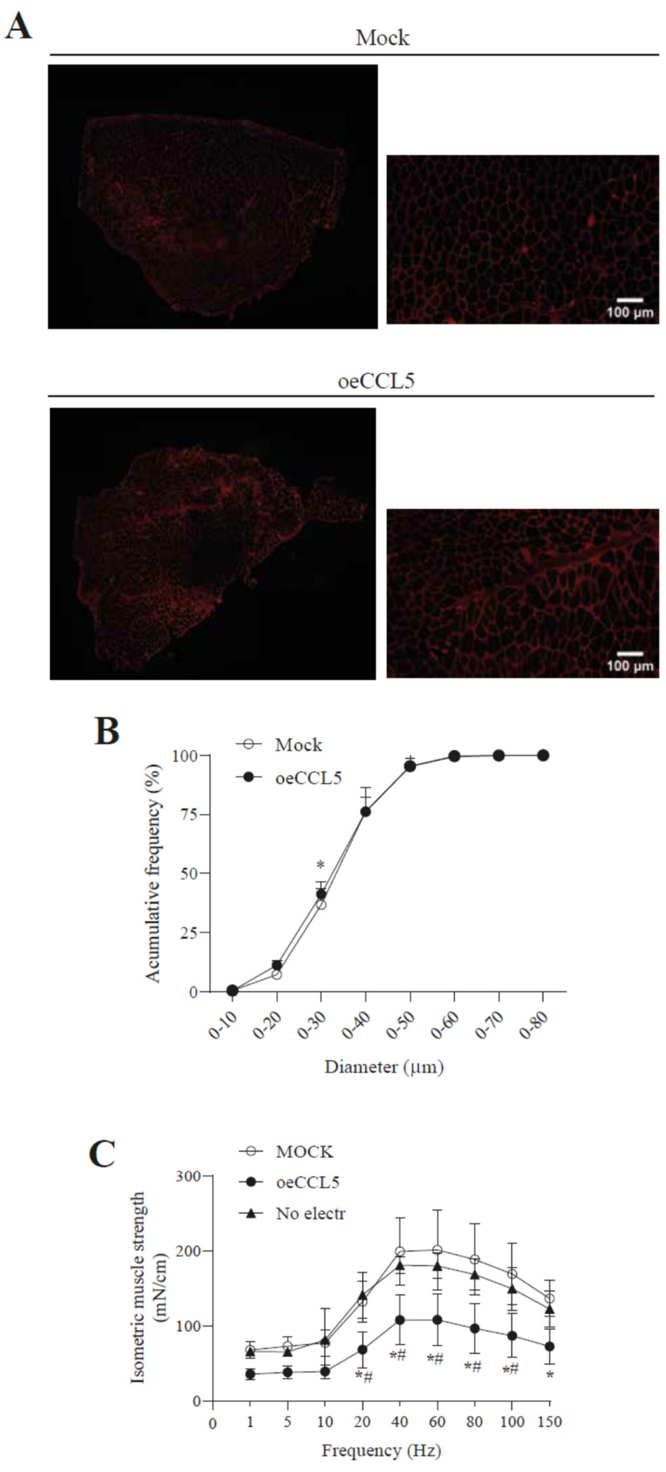

Supplementary Figure 3

Supplementary Figure 3. Overexpression of CCL5/RANTES in TA does not produce sarcopenia at 7 days post electroporation. TA muscles from C57BL/6 male mice were electroporated with a control plasmid (Mock) or a plasmid overexpressing CCL5 (oeCCL5). **(A)** TA muscles were removed 7 days post-electroporation, and cryosections were obtained (10 μm). **(B)** A cumulative frequency graph of fiber diameters is expressed in % corresponding to a range of diameters. **(C)** Measurement of isometric force in TA, using 1 – 150 Hz frequencies and normalized for tibia length 21 days post-electroporation in no electroporated (No electr), Mock, and oeCCL5. Mean ± SD for each group (n=3-4 mice per group), t-test, *p<0.05vs. Mock. # p<0.05vs No electr.

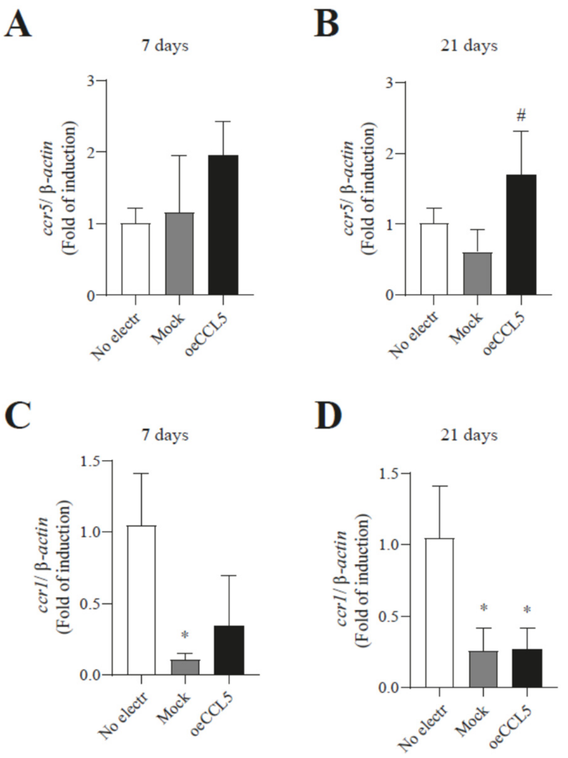

Supplementary Figure 4

Supplementary Figure 4. CCR1 and CCR5 expression under overexpression of CCL5/RANTES in TA muscles. TA muscles from C57BL/6 male mice were electroporated with a control plasmid (Mock) or a plasmid overexpressing CCL5 (oeCCL5). No electroporated muscles are also shown (No electr). CCR5 **(A, B)** and CCR1 **(C, D)** gene expression was detected in samples of TA muscles at 7- and 21-days post-electroporation through RT-qPCR using β-actin as a housekeeping gene. Mean ± SD for each group (n=3-4 mice per group), t-test, *p<0.05vs. No electr. # p<0.05vs Mock.

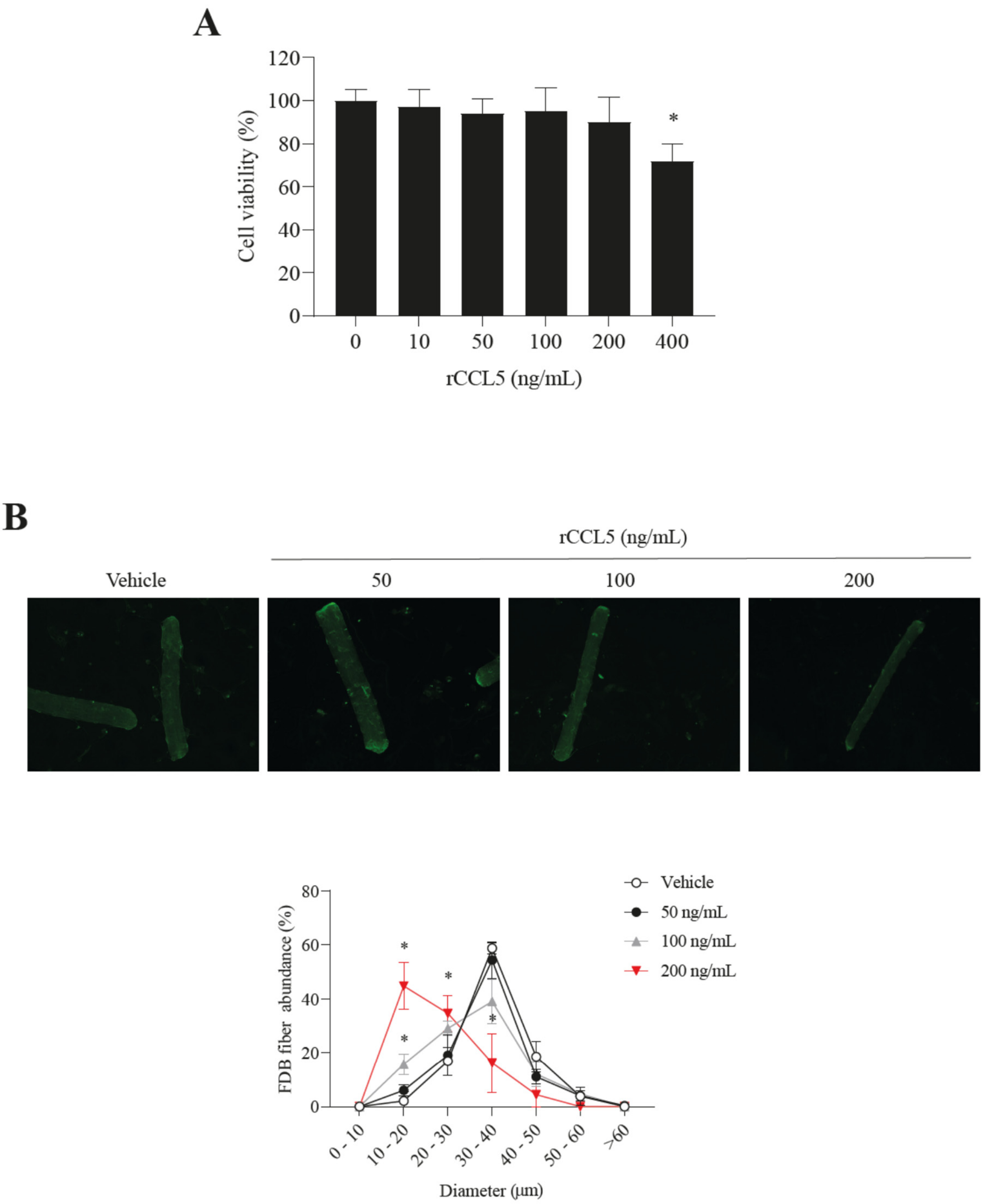

Supplementary Figure 5

Supplementary Figure 5. Dose-dependent effect of CCL5/RANTES in muscle cells. C_2C_{12} myoblast cell line, differentiated for 5 days into myotubes and muscle fibers isolated from FDB, incubated with increasing concentrations of CCL5/RANTES (0 up to 400 ng/mL) for 72 hr. h. **(A)** Cell viability was evaluated through MTT assay. **(B)** Delineation of muscle fibers isolated from FDB by immunofluorescence anti-Cav-3, magnification corresponding to 20X, and scale bar corresponding to 100 μm. Quantification of the diameter of FDB muscle fibers by abundance plot expressed in % relative to muscle fiber size ranges (μm). The results are expressed as the mean ± SD (n=3). 50 fibers were analyzed per condition for each "n" (a total of 150 fibers for each condition).

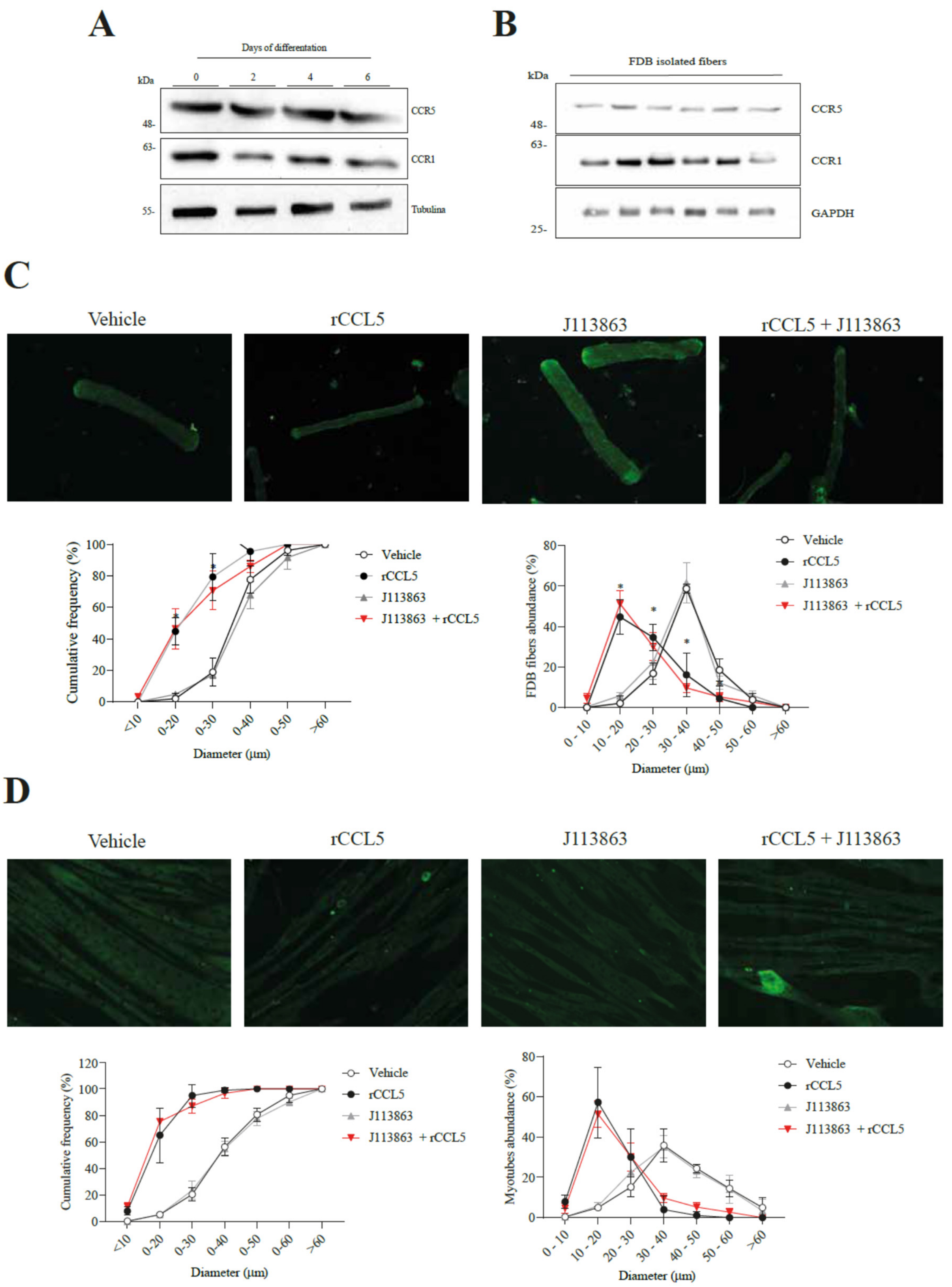

Supplementary Figure 6

Supplementary Figure 6. Presence of CCR1 and CCR5 receptors in muscle cells and participation of the CCR1 receptor in the decrease in the diameter of muscle cells treated with rCCL5. C_2C_{12} myoblast cell line, differentiated for 5 days into myotubes and muscle fibers isolated from FDB, were pretreated with 10 μM of the CCR1 inhibitor for 1 h and subsequently treated with 200 ng/mL of rCCL5 for 72 h. **(A)** Presence of CCR1 and CCR5 receptors in C_2C_{12} myotubes during the differentiation process into myotubes. **(B)** Presence of CCR1 and CCR5 receptors in isolated muscle fibers of FDB. **(C, D)** Surface delineation of C_2C_{12} myotubes by immunofluorescence against Cav-3, magnification corresponding to 20 X, and the scale bar corresponds to 100 μm. **(C)** Quantification of the diameter of C_2C_{12} myotubes, using an abundance graph expressed in % vs. range of myotube diameters and graph of cumulative frequency expressed in % of C_2C_{12} myotubes vs. diameter. **(D)** Quantification of the diameter of FDB muscle fibers by abundance plot expressed in % relative to muscle fiber size ranges (μm). and diameter quantification plotted as cumulative frequency (%) versus diameter. The results are expressed as the mean ± SD (n=3). 80 myotubes and 50 fibers were analyzed per condition for each "n" (a total of 240 myotubes and 150 fibers for each condition). ANOVA two-way post hoc Bonferroni *$p<0.05$ vs Vehicle.

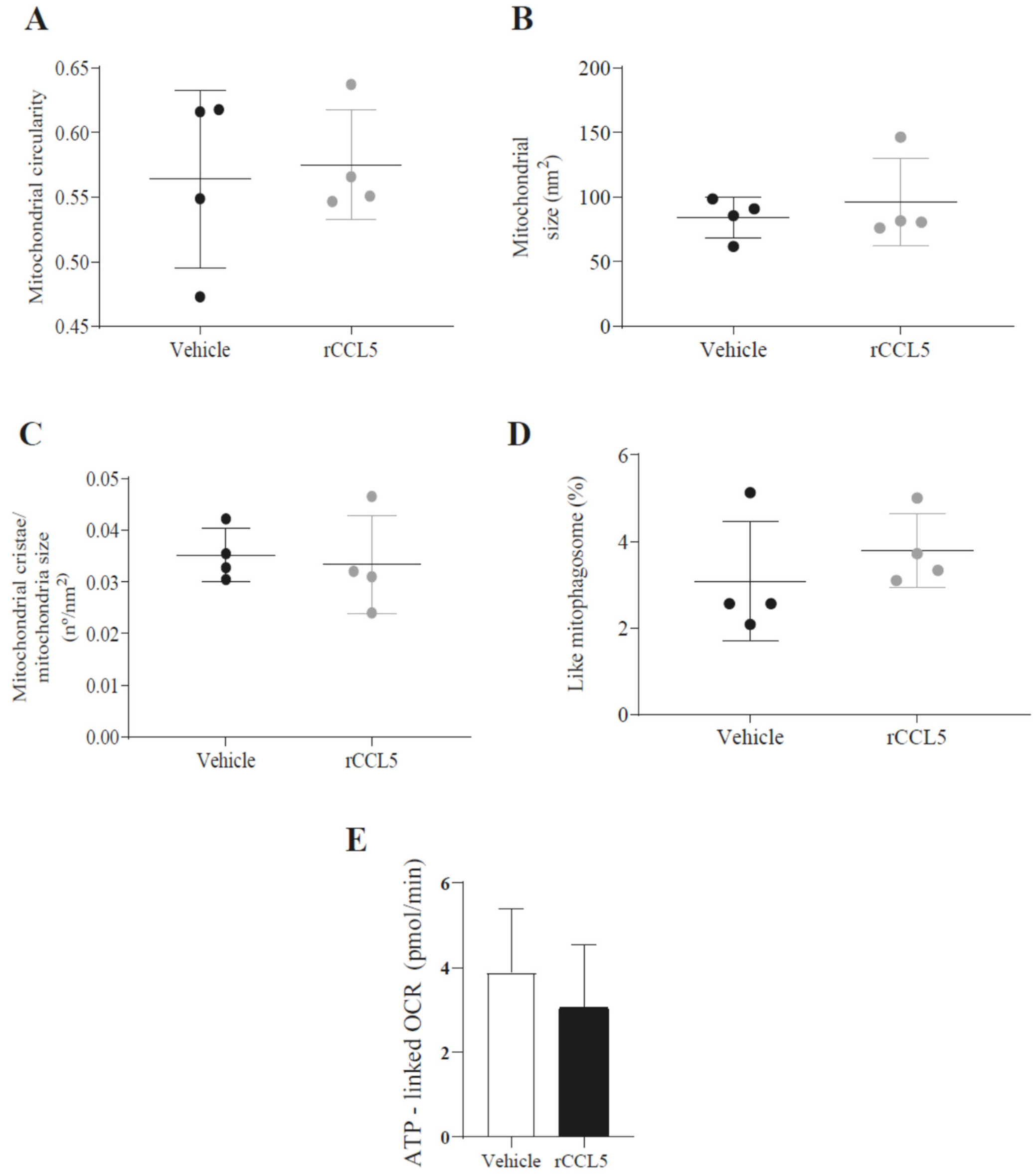

Supplementary Figure 7

Supplementary Figure 7. CCL5 does not alter the structure of mitochondria, nor does ATP-linked OCR. C_2C_{12} myotubes were incubated with recombinant CCL5 (rCCL5) at 200 ng/mL for 72 h. Representative mitochondrial images. For each independent experiment, mitochondria of at least three fields in four myotubes were counted as 6–8 mitochondria per field **(A)** Mitochondrial circularity was determined as width/lengthy ratio, **(B)** Mitochondrial size in nm2, **(C)** the number of mitochondrial cristae was normalized by mitochondria size after incubation with rCCL5. **(D)** Detection of mitophagosome-like structures expressed in %. **(E)** ATP-linked OCR (pmol/min) was determined by OCR analysis using Seahorse. The results are expressed as the mean ± SD (n=3 t-test)

A

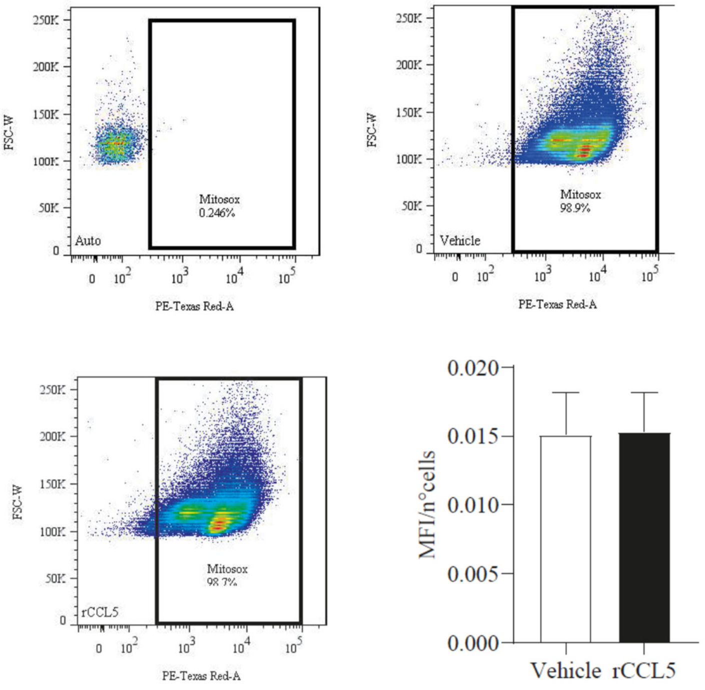

B

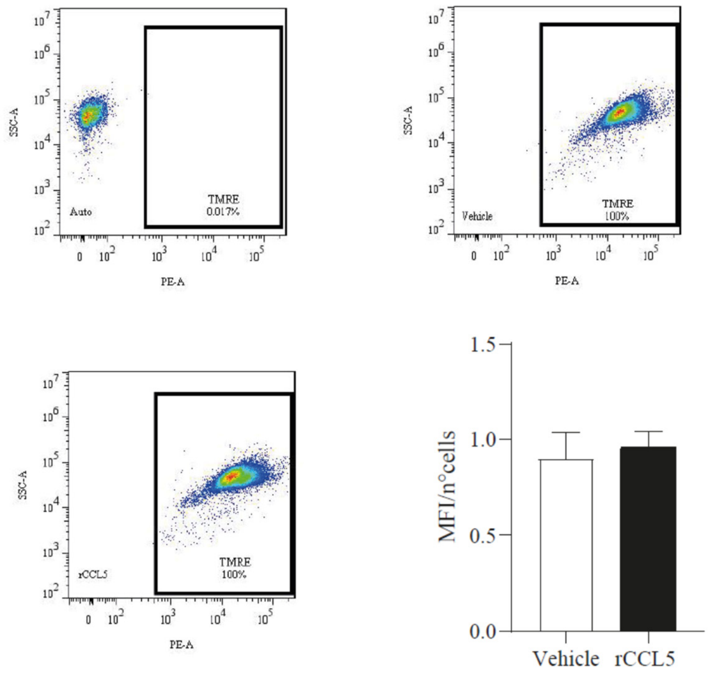

Supplementary Figure 8

Supplementary Figure 8. CCL5 does not alter mitochondrial functionality. C_2C_{12} myotubes were incubated with recombinant CCL5 (rCCL5) at 200 ng/mL for 72 h. Myotubes were incubated with **(A)** 10 μM of MitoSOX and **(B)** 400 nM TMRE probe for the detection of mitochondrial ROS and mitochondrial membrane potential for flow cytometry analysis. Representative flow cytometry dot plot analysis is used to identify mean fluorescence intensity (MFI) for each condition. Analysis of the MFI normalized by the number of cells (n°cells) in myotubes incubated with rCCL5. Values are expressed as the mean of MFI/n°cells ± SD (n = 3 independent experiments).
